# Correction: Visualizing conformational dynamics of proteins in solution and at the cell membrane

**DOI:** 10.7554/eLife.44029

**Published:** 2018-11-30

**Authors:** Sharona E Gordon, Mika Munari, William N Zagotta

Gordon SE, Munari M, Zagotta WN. 2018. Visualizing conformational dynamics of proteins in solution and at the cell membrane. *eLife* **7**:e37248. doi: 10.7554/eLife.37248.

Published 20, June 2018

During final figure preparation, the similar graphs in Figure 10C, D and Figure 11C, D were accidentally switched, a mistake that was noticed by the authors when rereading the published version. We have moved the graphs to the correct figures. This correction does not affect the results or conclusions of the original paper.

The corrected Figure 10 is shown here:

**Figure 1:**
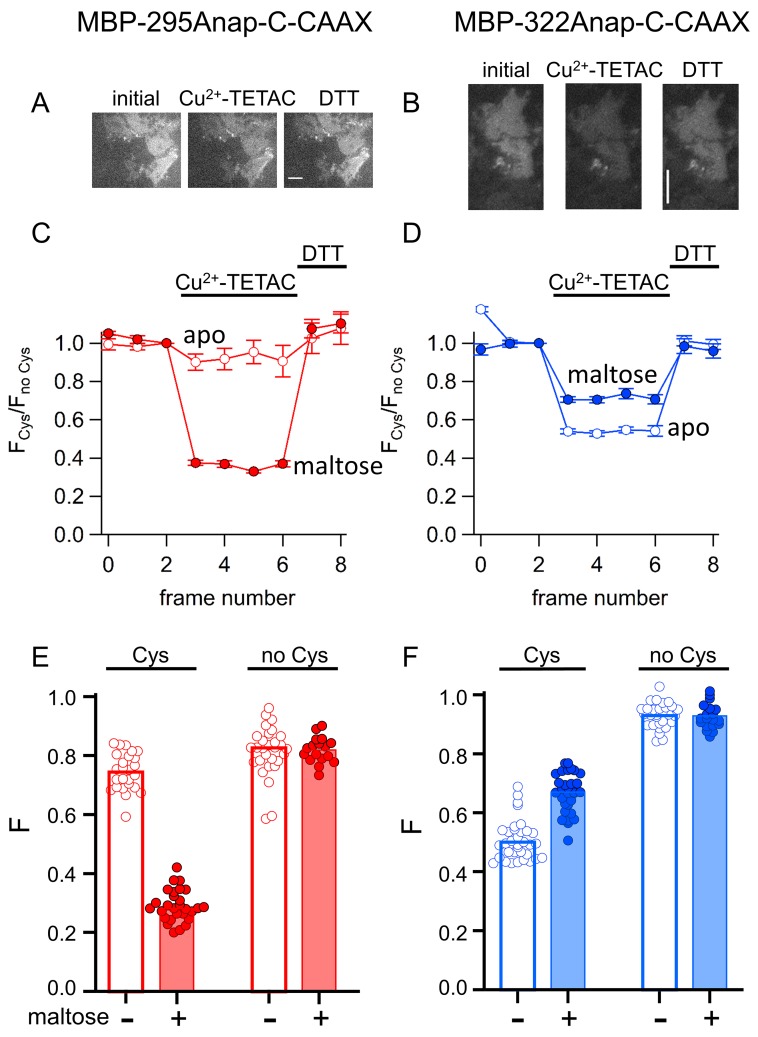


The corrected Figure 11 is shown here:

**Figure 2:**
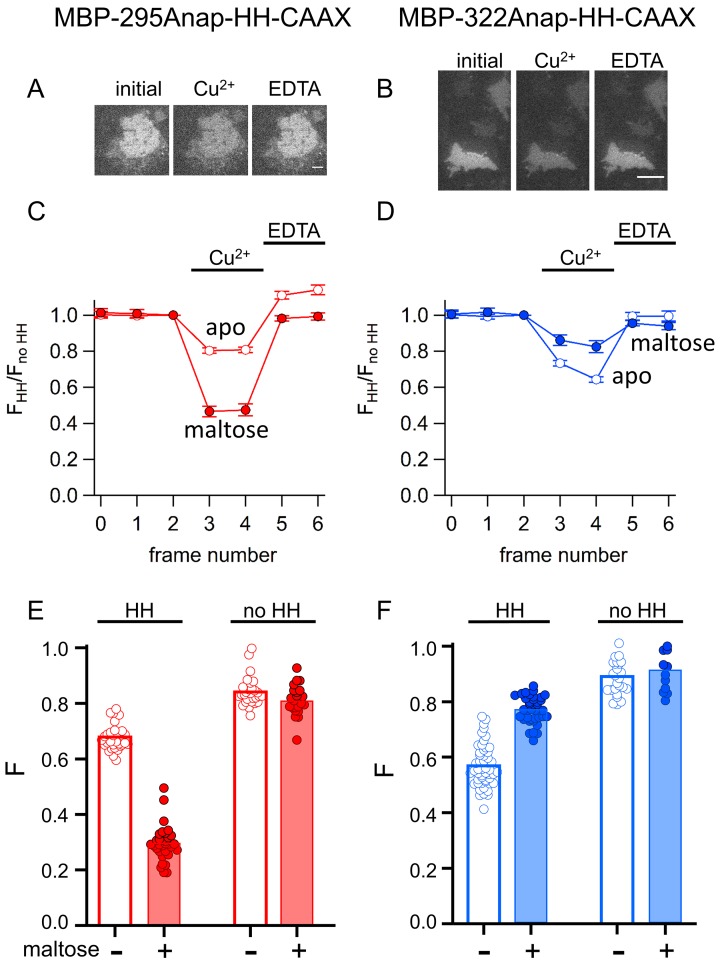


The originally published Figure 10 is also shown for reference:

**Figure 3:**
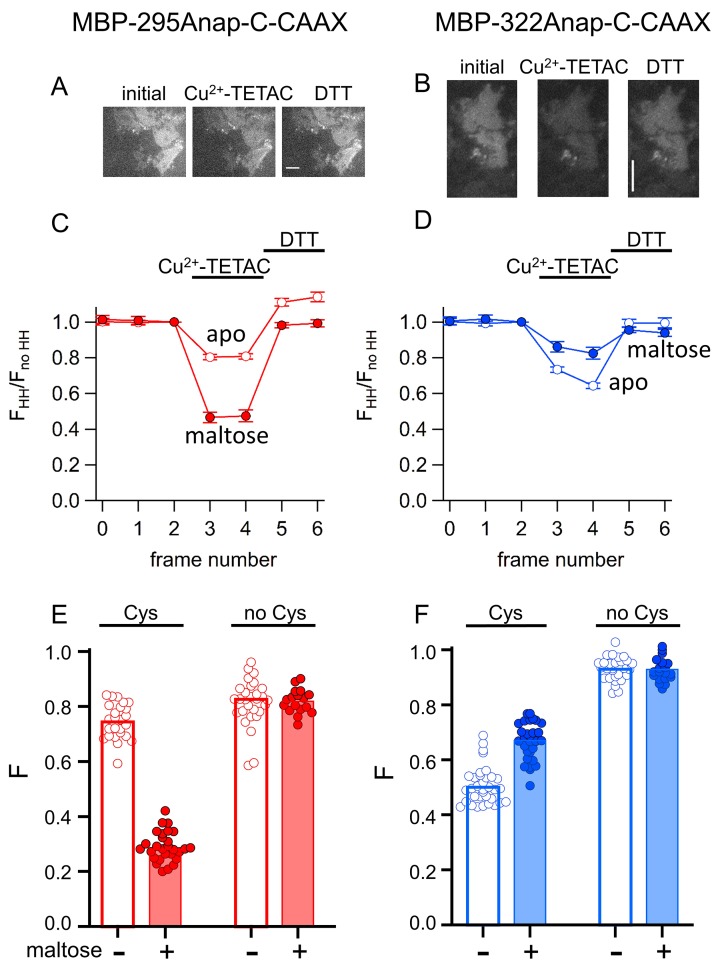


The originally published Figure 11 is also shown for reference:

**Figure 4:**
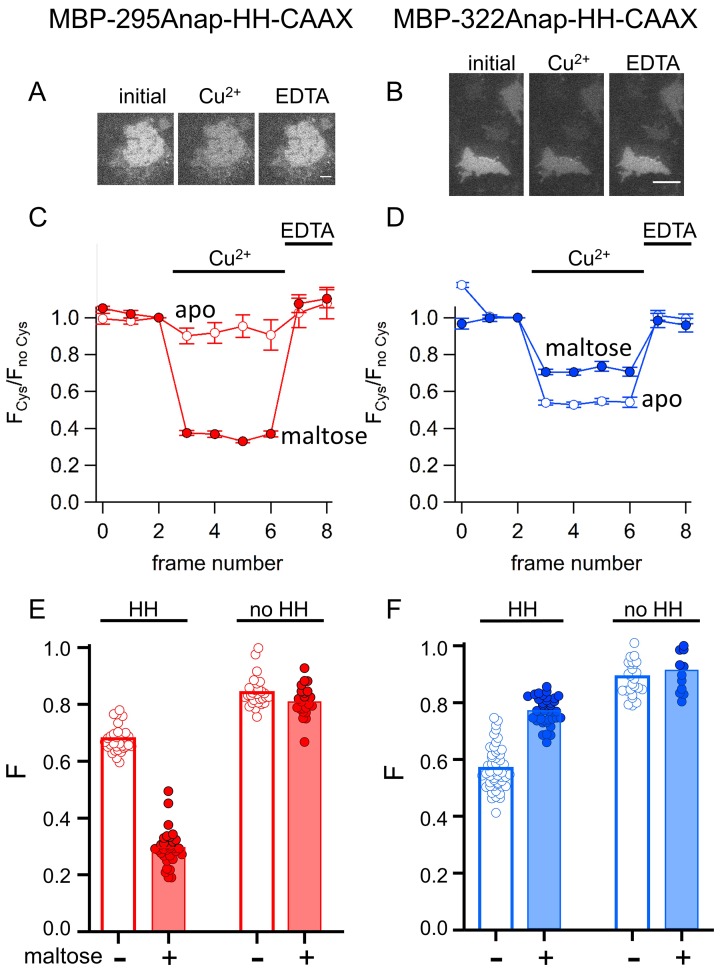


The article has been corrected accordingly.

